# Novel Pumping Methods for Microfluidic Devices: A Comprehensive Review

**DOI:** 10.3390/bios12110956

**Published:** 2022-11-01

**Authors:** Aleksei P. Iakovlev, Alexander S. Erofeev, Petr V. Gorelkin

**Affiliations:** Research Laboratory of Biophysics, National University of Science and Technology «MISiS», 119049 Moscow, Russia

**Keywords:** microfluidics, active pumping methods, passive pumping methods, point-of-care devices, lab-on-a-chip, lab-on-a-disk

## Abstract

This review is an account of methods that use various strategies to control microfluidic flow control with high accuracy. The reviewed systems are divided into two large groups based on the way they create flow: passive systems (non-mechanical systems) and active (mechanical) systems. Each group is presented by a number of device fabrications. We try to explain the main principles of operation, and we list advantages and disadvantages of the presented systems. Mechanical systems are considered in more detail, as they are currently an area of increased interest due to their unique precision flow control and “multitasking”. These systems are often applied as mini-laboratories, working autonomously without any additional operations, provided by humans, which is very important under complicated conditions. We also reviewed the integration of autonomous microfluidic systems with a smartphone or single-board computer when all data are retrieved and processed without using a personal computer. In addition, we discuss future trends and possible solutions for further development of this area of technology.

## 1. Introduction

Microfluidics (microhydrodynamics) is the science and technology of systems that use and study the properties of liquids limited by small volumes of fluids (of the order of 10^−9^/10^−18^ L), in channels ranging in size from tens up to hundreds of micrometers [1]. Manipulating small volumes of fluids allows for combining different processes, automating them, and integrating into complex systems [2,3,4]. Such systems are superior to larger ones, with higher control over spatiotemporal dynamics, as well as low fluid flow and ease of use. These systems provide very high efficiency and repeatability [5,6,7,8]. All listed characteristics make microfluidic systems a great tool to carry out a cascade of analytical operations inside a single device. Hundreds of simultaneous biochemical and biophysical experiments can be performed in a branched network of microchannels formed on a single substrate (usually PDMS—polydimethylsiloxane) with high efficiency. This type of device is called a biochip (Colyer et al., 1997) [9] or Lab-On-a-Chip (LOC) [10]. The main attractiveness of these platforms is the ability to perform a wide range of different analyses at the same time within one device with high efficiency.

In recent years, microfluidic devices have been increasingly utilized for different biophysical tasks, such as cell cultivation [1,2,3,4,5], cell separation [6,7,8,9,10,11], expansion of CTC from patient blood [12,13,14,15,16,17], flow cytometric analysis and cell manipulation [18,19,20,21,22,23], tissue engineering (Organ-On-a-Chip) [24,25,26,27,28,29], cell sorting and cell counting [30,31,32,33], and point of care (POC) diagnosis [34,35,36,37,38,39]. Many biochemical analyses can be screened at a faster rate in disease diagnosis [40,41]. Microfluidic systems also find their application in the fields of foodborne pathogens detection [42,43,44]. Low costs for all materials, simplicity, and low reagent consumption allows for fabricate cost-effective disposable and for mass production [45].

The materials used for microfluidic devices can be divided into five groups [46,47]. The first group includes elastomers, polymers, PDMS, and polyester [48,49]. The second group consists of molded thermoplastics, and the third one includes thermosetting polymer processed by photopolymerization [50,51,52]. The fourth one contains paper, which is often used for disposable systems and POC systems [53,54]. The fifth group includes hydrogels, which are widely used in biological applications related to living cells [55,56]. The most significant problem in microfluidics is the method of moving the reagent through the channel network. Flow generation systems can be divided into two large groups: passive flow generation systems and active systems [57,58]. Active systems typically include solenoid valves, pumps, stepper motors, micropumps, magnetic, electric, or thermal forces [59,60,61,62]. Passive systems move the liquid through the channels of the chip under the action of external forces, such as: gravitational, capillary, and surface tension, without additional power supply [63,64,65].

Microfluidic fluid flow can be characterized by the following parameters: density, viscosity, velocity gradient, and the Reynolds number [66]. Users can control the fluid flow rate and its direction by varying these parameters. The geometry of a channel and the physical properties of liquid can also be exploited to provide additional flow control. Different types of microfluidic systems require various mechanisms of flow-driven forces as well as different variations of microchannel parameters. Due to the incompressibility of the liquid, the flow in microsized channels has a small Reynolds number, usually less than 1, and the flow in simple microchannels is laminar; thus, chaotic or turbulent flows are not observed [67]. This flow behavior is proposed to develop various types of microfluidic devices.

In this review, we focus on the currently existing systems for creating flow inside the microfluidic chip and compare them.

## 2. Passive (Non-Mechanical Systems)

Passive flow control methods usually rely on a microchannel structure or natural effects; they do not require complicated mechanical parts and external power sources, which means that passive driven systems can be employed for low-cost and portable microfluidic applications; hence, they can be driven almost everywhere.

Passive flow control methods can be divided depending on the force that creates the flow: gravity-induced, capillary action, surface tension, vacuum suction, osmosis, pressure-driven. Further in the text, we discuss all these techniques and their pros and cons.

### 2.1. Gravity-Driven Flow

Gravity force is one of the simplest techniques used to create a slow flow rate in microfluidic systems. The main challenge for this technique is the small volume of samples, which makes the gravity of liquids negligible compared with other forces such as surface tension. Different combinations with other methods or smart designs of microfluidic chips have bypassed this problem.

In the simplest case, the gravity-driven method is based on a “system” of communicating vessels: the system consists of two reservoirs filled with liquid, located at different heights [68,69,70]. Fluid flow is driven by the Earth’s gravity, and the flow rate depends on the viscosity of the fluid and the height difference between the inlet and outlet of the microfluidic device.

**Figure 1:**
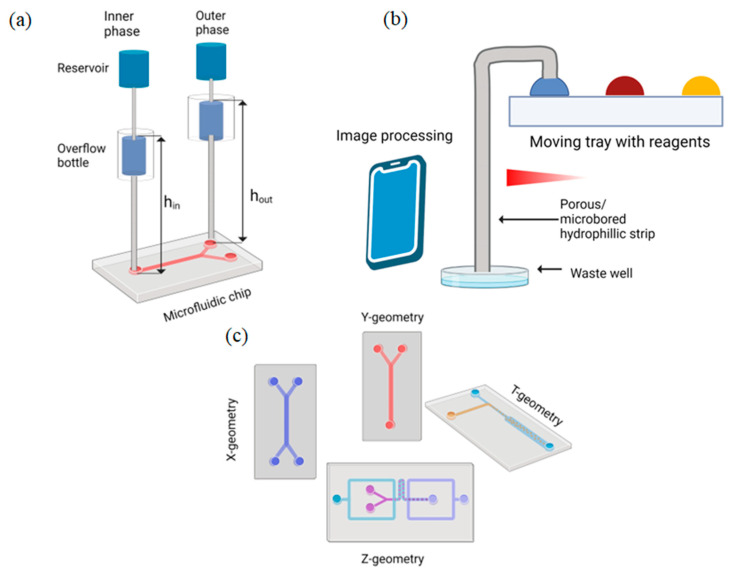
Schematic illustrations of gravity-driven systems: (**a**) semi-open gravity-driven overflow microfluidic flow supply system; (**b**) gravity-driven microfluidic siphon; (**c**) stand-alone pressure-driven 3D microfluidic chemical-sensing analytic device with different channel geometries.

Gao W. et al., presented a semi-open gravity-driven overflow microfluidic flow supply system for the generation of monodispersed droplets [71]. The systems consist of two independent injection units and a microfluidic chip. Each injection unit in turn includes a semi-open liquid reservoir and an overflow bottom, located below the reservoir. The inlet of the overflow bottle connects to the reservoir, and the outlet leads to the microfluidic chip. Figure 1a illustrates the schematics of the microfluidic flow supply system. The flow stability was verified in comparison with a syringe pump system by tracking the two-phase flow interface with low interfacial tension.

Reis N. M. et al., demonstrated the gravity-driven microfluidic siphon, made of three different materials: a strip of porous membrane, single-bore glass capillaries, or microbored material [72]. The operation of a siphon is only dependent on the hydrostatic liquid pressure and not the capillary forces, which result in zero dead volume in the device and no reagent overlap or carryover. Figure 1b illustrates a working principle of the device. The siphon concept can be transformed to POC devices as a new generation of power-free flow driving methods.

In 2016, Shin J et al. designed a stand-alone pressure-driven 3D microfluidic chemical-sensing analytic device (PD-PAD) [73]. The authors designed microfluidic chips with a varying geometry of channels (T, X, Y, Y2, and Z), but the whole principle of fluid flow was the same: the height difference between the inlet and outlet of the device. Figure 1c illustrates the geometry of the microfluidic channels. The authors compared flow rates of the fluid depending on different geometries of the channels and showed that a stand-alone pressure-driven microfluidic device has a great opportunity to be used as POC diagnostics.

Yu-Ting Kao presented a stand-alone microfluidic system that allowed for the execution of digital enumeration of bacteria and digital antibiograms without any specialized microfluidic instrumentation [74]. The authors compacted all supply channels onto the chip, unlike in previous developments [75,76]. The developed system contained an oil reservoir with an inlet, four sample inlets and chambers, four droplet chambers, and two oil outlets, which connect two droplet chambers. The oil flows only when both the oil inlet and the oil outlet are open. After orienting the device vertically, liquid through the step emulsification module is driven by pressure above 4.73 mbar, which arises from the height difference of 30 mm between the oil inlet and the oil outlet. The authors showed good stability of the droplets and the ability of the platform for long-term culture of bacteria.

#### Limitations

Gravity-driven microfluidic systems can only generate continuous flow [77], which may not be applied for specific cell culture applications, where dynamic or pulsatile flow is necessary. This technique also requires constant solution replenishment to maintain the continuous flow, i.e., such systems cannot be applied for long-term usage.

### 2.2. Capillary Action Flow

Capillary action is often applied in microfluidics to draw liquid autonomously in a substrate without the need of any external energy such as pumps or external pressure. This effect takes place due to the surface tension and the wetting properties of the capillary, which overcome the effect of gravity and viscosity of the liquid and is defined as the movement of a fluid within the spaces of a porous material due to the intermolecular forces between the liquid and the surrounding solid surfaces. Surface properties of the channel, the type and viscosity of the fluid, and the internal geometry of the capillary—all these factors affect the capillary flow and its velocity. One of the distinguishing features of such a ‘pumping’ method is the stable flow rate and stopping filling the capillary without the need for external control. All listed factors allow building independent system for fast analysis.

So-called capillary pumps have been widely used for different types of applications [78,79]. There are various modifications of these devices [80], and some of the previous devices are still in use [81]. Epifania R. reported the single-step capillary-based microfluidic device for sub-minute detection of analytes by a fluorescent competitive immunoassay. The authors determined the minimum detectable limit of the device—1.7 ng/mL using 4.5 µL of sample [81]. The device consists of two main elements: the chamber with packed beads and the capillary pump. Capillary pump which drives the fluid through the microfluidic device was made up of two segments: first one is a 22 mm long and 300 µm wide channel, which is followed by 115 mm long one, with walls composed of 150 × 50 µm pillars spaced by 75 µm gaps. The first segment accumulates hydraulic resistance. The second one allows the liquid to move along the main channel, and the gaps spaced in it allow to keep constant flow rate. The obtained results highlighted the developed device as a potentially multiplexable device that is generally used to perform rapid analyses of multiple analytes in POC diagnostics.

Capillary-driven systems are often used in the sphere of detection of proteins or as indicators for chemical reactions. Hassan S. et al. moved further and united smartphone and capillary-based microfluidic device. The authors reported on the device, which works on the principle of capillary-driven flow microfluidics and allows detection by multiple microchannels in a single microchip via smartphone imaging [82]. Fabricated microchannels of the device have the following dimensions: 8 cm long, 0.25 × 0.20 mm (width × depth). The mean flow velocity inside such a system is 15 mm/s. The chip was fulfilled by the sample via capillary action after placing the device with prepared into microchannels reagents into the sample solution. The device was left in incubation to allow reaction to occur and produce colorimetric signal. Finally, the photo of the device was captured by smartphone, for further analysis via image processing.

Ahi E. E. et al., continued their previous work and developed sandwich-based microfluidic chip-based immunoassay device to detect Human Chorionic Gonadotropin (hCG) protein [83]. The authors showed that their device minimizes the consumption of reagent and sample and allows rapid progress of immuno-assay procedure in the microfluidic chip. The chip was composed of 6 successive chambers connected via microchannels less in depth than chambers. The chambers have one input port and one outlet port for loading the samples or reagents and venting air, respectively.

#### Limitations

It is too complicated to satisfy all parameters, such as surface tension, geometry of the channel, viscosity of the fluid, and adhesion of the analyte to the channel surface, to achieve a certain flow rate value in capillary-driven systems. The channel surface of the device needs be covered by surfactant with multistep procedures. The concentration of the liquid in the outlet reservoir also limits the overall flow duration through the entire chip.

### 2.3. Surface Tension Flow

The cohesive forces between the liquid molecules are responsible for the phenomenon known as surface tension [84]. This phenomenon is defined as a property of the surface of a liquid that allows it to resist external forces. In microfluidics, different variations of surface and liquid allows for controlling passive driven flow.

**Figure 2:**
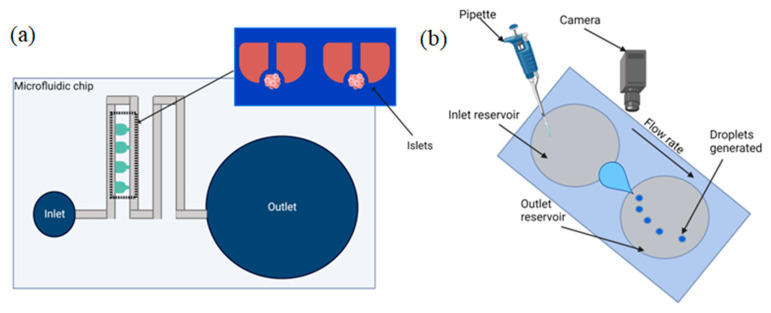
Schematics of microfluidic systems with surface tension flow rate regulation: (**a**) microfluidic array to study Langerhans pancreatic islets; (**b**) schematics of droplet microfluidic system.

Xing Y. et al., in 2016, presented a pumpless microfluidic array driven by surface tension to study Langerhans pancreatic islets [85]. Figure 2a illustrates the schematic of the proposed device. The driven force occurs in response to the inlet and outlet size differences, which cause a surface-tension flow through the system of microchannels. There is an islet array between the inlet and outlet that holds 20 individual islets based on the hydrodynamic trapping principle.

De Groot T. E. et al., presented an open hanging droplet culture platform. This device has lower shear stress compared to the analogues system, which is achieved through the use of two interconnected hanging droplet wells. The device consists of two connected and open droplets: a culture droplet and a user interface droplet. System openness can add or remove culture dynamically during the experiment [86]. They showed that an asymmetric two-well droplet system enables the long-term culture of shear-sensitive cells.

Calver S. N. et al., using matched asymptotic expansions, calculated the variable flow rate depending on the geometry of the microfluidic channel. The authors developed a model for the flow through a microfluidic device driven by surface tension [87].

Khor J. W. with colleagues reported an open channel droplet microfluidic system that autonomously generates droplets by leveraging competing hydrostatic and capillary pressure. The authors offer enhanced usability, direct access to the droplet contents, easy manufacturability, compact footprint, and high customizability [88]. The device is split into five parts: the inlet reservoir, leading to the converging region, which is followed by the narrow constriction, the diverging region, and the outlet reservoir. A schematic of the system is illustrated in Figure 2b. The developed system showed a droplet generation rate of up to 2 droplets/second with a volume from 100 nL to 120 µL without intervention. Khor et al. developed a theoretical model to determine the conditions for droplet formation, and both the theoretical model and experimental results showed the importance of the interfacial tension, contact angle, and constriction in droplet formation.

In the following study, the authors presented a pump-free microfluidic device with capillary-driven flow and passive mixing system for the electrochemical determination of AGP (α1-Acid glycoprotein) [89]. The device was composed of four inlets, four microchannels that merge into a single channel, and one outlet. The key factor for controlling the flow rate is the channel height varying from 50 to 100 µm between different layers of the device. The proposed device can serve as a POC testing system for clinical analyses.

#### Limitations

Platforms that use surface tension to drive fluid through the chip require timely replenishment of the solution to keep flow rate constant; moreover, they force makers to carefully select materials to fabricate the devices with specific functions.

### 2.4. Vacuum Driven

Vacuum-driven systems have become widespread due to the simplicity and ease of their application. Devices with the vacuum-driven method use the gas permeability of PDMS to drive the liquid through the chip. Often, such a pumping method is used in combination with other passive methods, and it is widely used for sample loading, storing, mixing, cell separation and culturing.

Hu J. reported a vacuum-driven microfluidic array for multistep loading of a sample [90]. The system comprises a connection of multi-level bifurcation microchannels intertwined with 4096 dead-end microchambers for liquid partitioning. Gas permeability of PDMS is the main flow-driven mechanism in such a system. The authors showed the ability of the device to perform digital PCR assays, including single cell and single molecule analyses, and they demonstrated the ability for single bacterial cell detection.

In the following study, the authors developed a vacuum-driven power-free microfluidic chip for exosomal mRNA detection [91]. The chip was fabricated in a “Y” shape with a channel height of 0.1 mm and width of 0.6 mm. The vacuum-driven system of this device was based on the gas permeability of PDMS. The authors were assured that the chip can overcome the spatiotemporal limitations of other POC systems, because their device generates a high flow rate without any additional source of energy. This device can be high effective for cancer diagnosis and liquid biopsy applications.

Zeng W. presented a hand-powered vacuum-driven device to rapidly generate different concentrations of the sample in microfluidic chambers. Syringes with a size of 1 mL were used as the vacuum source [92]. The platform was designed in the following way: two rows of dead-end culture chambers (C-chambers) connect with the main channel via both side channels (six on each side) and 12 vacuum chambers (V-chambers) located at the outer side of each C-chamber. The C-Chambers and V-Chambers share the same dimensions of 300 × 500 × 30 μm. The stable pressure of such a system can pump antibiotic droplets into the dead-end C-Chambers for further dilatation, which forms a concentration gradient of antibiotics. The developed system showed great high-output potential, with eight sets of AST (antibiotic susceptibility testing) assays performed simultaneously. The authors planned to unite smartphone-based colorimetric detection with their device as a further development of the system.

The following device is a combination of two types of flow-driven methods [93]. The authors used centrifugal force combined with vacuum-driven force to drag the sample through the PDMS microfluidic chip. Centrifugal force was used for sample loading and mixing, when the vacuum-driven force prevents the formation of air bubbles. The device consists of five sets of metering and dilution chambers corresponding to serial dilutions with various concentrations and waste chambers, for holding excess volumes of the sample. Two vents were additionally used for waste chambers, and four others were used for chambers with different concentrations. The series of ten-fold serial dilutions was processed in the following way: the sample was transferred from the metering chambers to the dilution chambers, where the mixing was processed, along with one-tenth of the sample volume from one dilution chamber to the next dilution chamber. The key feature of the developed system is its ability to complement the drawbacks of each flow-driven method, which can effectively use suc h a system for applications relating to novel biological or chemical on-chip analyses.

#### Limitations

The most common drawback of such systems is the low flow rate that arises from limited air diffusion and hydrophobicity of the PDMS material. Systems with vacuum-driven control can commonly be used only once, which makes them difficult to use for long-term experiments.

### 2.5. Osmosis

Osmosis is the tendency of molecules (usually water molecules) to pass through a semipermeable membrane from the region of less solution concentration into a more concentrated one [94]. The liquid movement stops when the concentration of the two sides reaches equilibrium. Osmotic-driven flow occurs when the draw solution contains more solute than a feed one, which causes higher osmotic pressure.

In the following study, the authors developed a fuel cell system that operated with an osmotic pump for a long period [95]. The osmotic pump has a cylindrical shape with a diameter of 30 mm and a height of 15 mm, and the larger diameter of the pump affects the duration of the generated flow (in that study, the duration was ~1 day).

Chuang C. H. and Chiang Y. Y. presented a novel version of the osmotic pump [96]. Regulation of the flow rate was implemented by controlling the osmotic area and the concentration of the draw solution. In this study, the authors introduced a new concept of Bio-O-Pump: a way of creating fluid flow with the help of a semipermeable biomimetic membrane containing water-channel proteins, aquaporins. This strategy can easily manipulate with volumes from a few microliters to milliliters by controlling the osmotic area and the concentration of the draw solution. The device was designed in two variants: with ψ- and vine-like channels, and it continuously generated liquid flow rate through the channels up to 4.88 mL/h. The authors additionally demonstrated the ability of the Bio-O-Pump fluid delivery strategy for chemical mixing and single-cell positioning applications.

#### Limitations

An osmotic-driven system requires more complicated structure than surface-tension, capillary, and gravity-driven devices. Such systems also have unstable and inaccurate flow rate.

### 2.6. Pressure-Driven Systems

The feature of pressure in liquids that is equally divided in all directions allows to manipulate big volumes of the liquid through a small input force [97]. In such a technique, the pressure driving force is conducted in the reservoir, for further driving the sample through the network of microfluidic channels.

**Figure 3:**
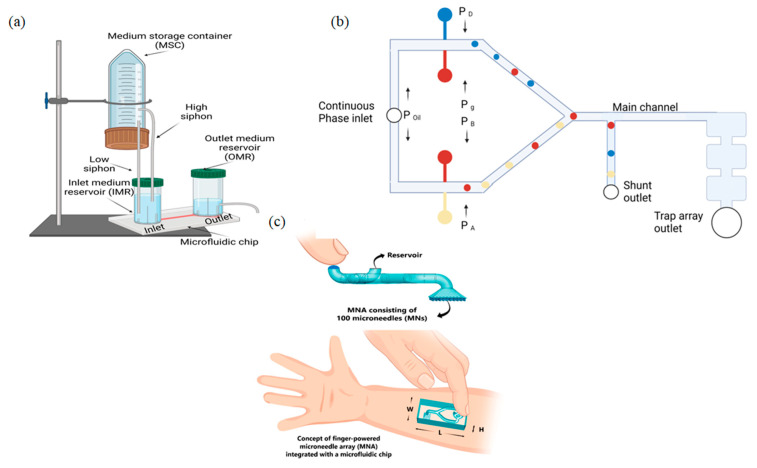
Examples of pressure-driven systems: (**a**) pump with siphon-based autofill function; (**b**) pressure-driven droplet generating system; (**c**) finger-powered microneedle array combined with microfluidic chip (from M. R. Sarabi et al. [99]).

A collaborative team from China and the United States of America solved the issue of steady and continuous flow for passive pressure-driven micropumps by improving the pump with a siphon-based autofill function [98]. The developed device can consistently provide steady medium perfusion inside the microfluidic chip for up to 5 days without the need for supplemental medium changes. The entire platform consists of four main elements: medium storage container (MSC) with large volume (e.g., 50 mL), inlet medium reservoir (IMR), outlet medium reservoir (OMR), and microfluidic chip. Figure 3a shows the schematic of the described system. The MSC is positioned above the IMR and is connected to it through two plastic tubings with different heights, which are functionalized as siphons. To activate the siphoning effect, the top end of the low siphon should be positioned as close to the bottom of the MSC as possible. Liquid level control inside the OMR is acquired by a tube inserted at the desired height to divert the used solution to a waste container, which allows for steady flow inside the microfluidic chip. The developed device can be used for cell or tissue culture inside the incubator for a long period and can be applied in systems such as Organ-On-Chip.

Schimel T. M. et al., reported a pressure-driven microfluidic droplet system to automatically generate arrays of complex droplet interface bilayer networks, providing a model of a cell membrane [100]. The microfluidic device was designed as a continuous phase channel, splitting into two channels at the inlet followed by two pairs of positioned streams, used for droplet production, and connected to the Y-junction, for further capture in an array of hydrodynamic traps. The schematic of the proposed system is shown in Figure 3b. The width and height of the main channel were the same across the entire device and are equal to 125 µm.

Zhang X. and Zhang Z. presented a passive flow regulatory device for enhanced flow control in microfluidics [101]. The device consisted of two functional parts: a flow regulating valve and a flow check valve. Such a configuration allows one to prevent backflow in the process of liquid delivery. The flow check valve includes a liquid chamber, an obstacle, a fluidic channel, and an elastic membrane with a hole. The flow-regulating valve included a shared fluid channel, a control channel, and an elastic membrane. The principle of operation of the device is to use a “feedback control system” consisting of valves, to prevent the liquid from flowing in reverse mode. The calculated flow rate for the system was 0.45 ± 0.2 mL/s.

Some of the pressure-driven systems may be classified as a separate category such as “human-pumping systems”, because of the method of flow driving. One of the most popular techniques for operating such devices is finger-pressure flow [102], which is often used for POC and LOC systems, implying that a user can carry out the analyses by themselves without the presence of healthcare workers.

Sarabi M. R. et al., combined a microfluidic chip with a finger-powered microneedle array to extract body fluid samples [99]. The microfluidic system included 100 microneedle arrays consisting of a deformable dome-shaped chamber, actuated by finger force, and microfluidic chip containing inlet for pressure input and reservoir outlet. The working principle of the microneedle array is shown in Figure 3c. The authors calculated the amount of the extracted blood in grams in 5 s per needle and gained the result of 12.24 µL of human blood, which lies in the range of the previous cases [103,104].

In the following work, the authors demonstrated a finger-actuated microfluidic device (μFAchip) for DNA extraction, gLAMP, and optical detection of multiple pathogens [105]. The proposed device was fabricated with three layers that enabled finger-actuated operation for controlled sample mixing. It contained four chambers with a cross-shaped valve—the key element for flow control between upper and lower chambers. Loading the sample into the bottom reaction chambers is provided by manual finger presses to actuate the cross-shaped valve. Further detection of bacterial strains was provided via a digital fluorescence microscope, demonstrating a sensitivity of 1.6 cells.

#### Limitations

External pressure, especially the finger-driven one, might affect the accuracy and repeatability of the generated flow rate. In addition, some pressure-driven systems can only provide low-pressure input to the microfluidic chips, i.e., it cannot be applied in applications needing accurate high pressure or flow rate.

The passive flow control method is the simplest way used in autonomous systems. This method is extremely convenient for the rapid diagnosis of diseases since it is quite cheap and easy to use. Passive pumping techniques are distinguished by their simplicity and small size; in this case, the sample moves through the channels under the action of internal forces [106]. The flow rate can reach up to 100 nL/s. Passive-driven systems have become widespread among end users, as they are the most compact, functional, and highly targeted devices, allowing them to be used outside the laboratory. Such systems can be used to detect nucleic acids in infectious diseases, to detect biomarkers of ovarian cancer, and to provide cell separation and quantitative analyses; however, despite the advantages of this method, it has a number of disadvantages that limit its potential. Capillary flow strongly depends on the adhesion of the analyzed object to the channel surface and the surface tension of the object itself [107]; moreover, passive systems are designed as disposable devices, making it impossible to reuse them in the laboratory. The flow rate of such systems is always lower than the velocity of manual control systems, and even more so than mechanical systems.

Manual control systems, unlike the passive ones, allow the researcher to control the entire process, which makes it possible to conduct more complex studies. The flow rate in such systems can reach 1 μL/h. The results of the studies may vary depending on the researcher, despite the ease of use, since such a method requires high accuracy from the user side; therefore, the repeatability of studies conducted with human-controlled systems can be very low. Manual control systems do not allow for simultaneous analysis of several samples, which consists in their controlled movement along the channels of the chip, which also makes it not so flexible.

## 3. Active (Mechanical Systems)

Active (mechanical) systems are based on automatic syringe pumps, micropumps, electromagnetic and magnetic valves, vacuum pumps, thermal forces, and electrokinetic interactions, unlike passive ones. Active pumping methods are more complicated but have become more widely used due to more precise non-pulsative and continuous-flow control. The versatility of self-contained microfluidic systems can be increased by using additional mechanical miniature elements integrated into the chip, allowing for highly controlled multi-stage experiments that cannot be achieved with passive systems and manual devices.

The active pressure-driven systems rely on various ways of controlling and generating fluid flow via electromagnetic pumps, valves, or syringe pumps. There are some commercially used systems for active pumping: (Cetoni Nemesys syringe pump, ElveFlow pressure controller, Fluigent pressure controller system, Space Tango CubeLab); such systems are not described in this review.

Optical methods, which are widely used in microfluidics for determining the cell’s response to treatment, have some difficulties in real-time and high-spatial resolution imaging of the cell’s function. These limitations can be eliminated by uniting microfluidic platform with different analytical tools, such as scanning probe microscopy. Scanning ion-conductance microscopy (SCIM) with a confocal module can be applied as a system for nano-scale 3D printing, creating nano-arrays, cell translocation and studying local mechanical properties of living cells [108]. It combines both pressure- and electroosmotic-driven forces to move the sample inside nanocapillaries or microchannels of microfluidic devices. This method in combination with microfluidic platforms such as LOC and Organ-On-Chip can be effectively used for determining human drug responses in preclinical studies, but such systems are not described in this review.

### 3.1. Pressure-Driven Systems

The most widespread and easy-to-fabricate method of active pumping is the usage of syringe pumps, activated by stepper motors or more complicated drivers. This method allows one to build an inexpensive highly accurate microfluidic flow control system. A syringe pump has a simple working principle: an accurate amount of fluid is pushed by the plunger of a syringe pump, which is actuated by the nut and is driven by the rotation of the stepper motor.

Many researchers try to create their own syringe-based flow control systems to avoid the shortcomings of existing systems. Lake J. R. et al., developed a syringe pressure pump with feedback control to regulate the applied pressure inside microfluidic chip channels [109]. The system was designed and composed of four main components: the syringe pump, the pressure sensor combined with amplifier, the microcontroller, and the motor driver. The pressure sensor captures the value of the changing pressure when the syringe pump is displaced. Depending on the changing value of the pressure, the microcontroller outputs a signal to the motor driver, actuating the syringe pump, either increasing or decreasing the pressure. The authors implemented two control methods: bang-band and PID (proportional, integral, and derivative controller). Pressure signals were acquired using Arduino Uno (microcontroller-based platform), through the 10 bit ADC for every 200 ms.

A significant disadvantage of syringe pumps is their large overall dimensions compared to small micropumps. Zhang Y. and Tseng T. M. developed the novel standalone automated all-in-one portable microfluidic system (PAMICON) with innovative flow control method inside a single-layer microfluidic chip [110]. The proposed system is assembled in a small compact size and is based on a single board computer with a desktop-grade operating system, i.e., it does not require additional host devices to operate with the user. It provides on-board regulatable pressure, vacuum generation and control by using multiple miniature DC peristaltic pumps. The fluid control mechanism was built on the principle of valves, by injecting air or gas into the microfluidic channel. This injection can completely block, direct, or divide the flow inside the chip, causing the valve-based fluid control mechanism to be bypassed without direct deformation of the microfluidic channel.

Another valveless platform was presented by Hettiarachchi S. [111]. He designed and developed an active microfluidic droplet generator, based on the principle of flow-focusing geometry, where two immiscible fluids are connected and driven through a long narrow channel.

The fluid was pumped to the narrow channel via two submersible pumps attached to both phases. The flow rate was controlled by varying the supply voltage of the pumps, which was performed with the help of a microcontroller-based platform, H-bridge motor controller, and internal power supply.

Jõemaa R. et al. presented a portable all-in-one dual-channel microfluidic pressure pump, which can be customized via smartphone and web UI [112]. The developed device can be controlled with two-phase solutions and continuously pumped without refilling. Furthermore, it has a wireless remote control and is powered by battery for portability. The pumping device consists of the low-power 32 bit microcontroller, data-acquiring module, presented by a piezoresistive pressure sensor, and two individually drivable piezoelectric pumps. The device is powered by a Li-Po battery package that does not affect the control of the device over Wi-Fi while using a browser-generated web UI. The stability is maintained by a PID control system.

de Graaf M. N. S. et al., developed a PID software controller, which integrates constant pressure differences into a fluidic circuit board, for accurate multiplexing of perfusion experiments [113]. The system is composed of two pneumatic pressure controllers, a flow rate sensor and a microcontroller unit that process and transfer data via serial communication. Flow control is acquired by controlling the pressure difference of two sensors located at the inlet and outlet of the microfluidic chip. According to this difference, the microcontroller PID regulator corrects the output signal, which leads to a constant and continuous flow.

#### Limitations

The most significant drawback of syringe pump systems is their dimensions and unidirectional flow, although systems with electromagnetic micropumps and valves devoid of such shortcomings require more complicated control systems and higher power supply, and their price is higher than for similar syringe pump systems.

### 3.2. Centrifugal Microfluidics

Lab-On-A-Disk (LOAD) platforms use centrifugal force to move liquid through the channels, unlike most LOC platforms, which use external pumping mechanisms such as syringe pumps or electromagnetic pumps to drive the fluid through the chip. The operation technique is quite simple: the sample is directly injected into the inlet, then the LOAD is rotated, and the sample is driven through the system under the influence of centrifugal forces. One of the crucial advantages of this platform is that multiple tests can be provided on a single disk simultaneously.

Centrifugal microfluidics methods are often used for cell separation, enrichment, plasma separation, chemical lysis, or amplification of RNA. In the following article, the authors developed a portable bead-based integrated microfluidic platform for centrifugal loop-mediated isothermal amplification of viral RNA directly from heat-inactivated nasopharyngeal swab samples [114]. The proposed microfluidic device consists of two chambers: the bottom chamber contains all electronic components, and the top chamber consists of the blue laser diode, serving for signal acquisition. The entire platform is controlled by a microcontroller-based development board, which runs the motor to rotate the disk. The rotation speed is measured by the hall sensor and controlled by feedback. The proposed system achieves a sample-to-answer analysis time from the sample collection and detection for 1 h.

The authors of the following article developed an automated centrifugal microfluidic platform for rapid detection of the hepatitis B virus (HBV) from whole blood [115]. The all-necessary reagents for DNA extraction were prestored on the disk, which makes it completely automated. The disk provides all steps of HBV DNA detection from plasma separation from whole blood to the amplification of nucleic acids. Accurate speed control was carried out with a special controller, activating the servo motor, and an electromagnetic clutch was used to modulate the direction of the disk. A laser diode, which was fixed on the stepper motor, controlled 13 valves of the disk, serving as nanoheaters of the PCR chambers. The authors gained a total HBV DNA extraction time of approximately 15 min.

Chen et al. continued their work [116] and reported a centrifugal microfluidic platform with integration of microarray technology [117]. They offered a novel immunoassay system for rapid and automated methods for contaminant screening and quantification in milk. The authors achieved the simultaneous detection of six contaminants in milk within 17 min. The proposed system can be adapted to detect other proteins or small molecules to aid in food safety.

Another option of centrifugal microfluidic platforms is cell enrichment. A scientific team from Iran fabricated two different highly efficient centrifugal microfluidic devices for rare cell isolation [118]. The first design isolates cells through the inertial effects and bifurcation law. The second one uses two permanent NdFeB magnets to capture magnetically labeled cells. As a further step, the authors combined both methods to fabricate a hybrid microseparator to enhance the separation efficiency. The combined device was split into four sectors: non-target chamber, target-chamber, inlet-chamber, and magnet area. The disk was mounted on a rotational apparatus equipped with a PID speed controller and optical sensor for speed measurements.

Gowda et al. developed a proof-of-concept [119] portable pathogen analysis system for bacteria detection in water. The developed system was coupled with a portable “CD Driver” capable of automating the assay steps and providing single step bacteria detection on one device. The prototype system was tested to detect Enterococcus faecalis, a common fecal indicator bacterium in water samples. The device is a combination of droplet generation technology and loop-mediated isothermal amplification (LAMP) on microfluidic disks. The authors demonstrated the proof-of-concept of conversion of the binary LAMP results into a quantitative pathogen concentration through droplet digital LAMP. The microfluidic CD consists of four main layers. Bottom layer contains the major chambers and channels. Two middle biocompatible layers consist of the connecting channels. The top layer is a transparent polycarbonate disk with sample loading and vent inlets. The authors demonstrated the ability of the proposed device to integrate bacterial cell lysis in water samples, DNA extraction, and reaction droplet generation on a single disk practically without user handling.

Hwu et al. demonstrated reciprocation flow control through a reaction chamber on a centrifugal microfluidic device with bidirectional fluid flow accessed by only a rotating motor [120]. The proposed device consists of two main parts. The first one contains an inlet chamber, wide channels for fluid handling, receiving reservoir, microchannel, reaction chamber, pneumatic reservoir, and a gas compartment. The second one includes a reaction chamber for an array of heterogeneous immune assays integration. The authors characterized the CD liquid reciprocation by measuring the liquid levels. The simulation for determination of the flow velocities through the reaction chamber was also provided. These simulations could test two different liquid reciprocation profiles for a heterogeneous immunoassay.

**Figure 4:**
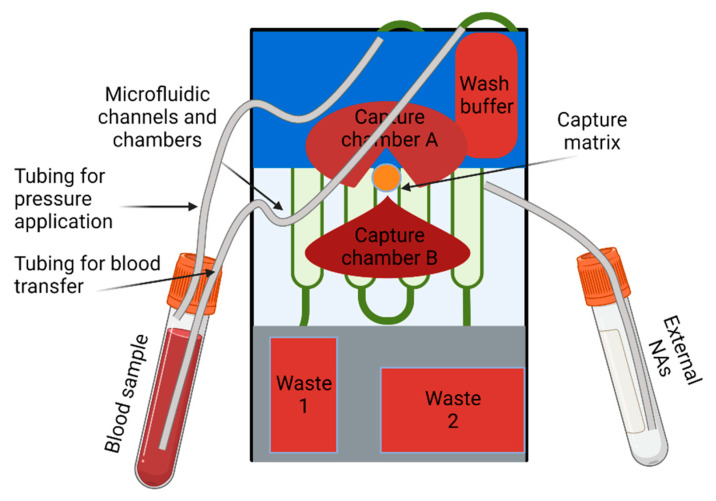
The schematic of the LOAD device combined with pneumatic valves.

Sometimes LOAD devices are equipped with other pumping methods. Thus, Brassard D. et al. combined pneumatic valves with a centrifugal microfluidic platform to perform an on-chip nucleic acid (NA) extraction from whole blood [121]. Combining the centrifugal-drive method with pneumatic actuation makes it possible to control the flow inside the chip without integrating any active components on the microfluidic platform itself. The design of the extraction cartridge implies the external vials for storing the blood and NA samples. The schematic of the proposed system is shown in Figure 4. These vials were used to simplify and automate the sample loading process inside the channels of the cartridge. The pneumatic valves apply pressure to the sample vial to transfer it to the cartridge. The vial with extracted NA samples fills with the same principal.

#### Limitations

Centrifugal microfluidic systems are complicated devices that require the centrifugal driven mechanism to rotate the microfluidic disk at a given speed. Such systems are very bulky and require complicated structure for the microfluidic chip to provide all required operations on it.

### 3.3. Electrokinetic Platforms

Electro-osmosis is one of the most popular active non-pressure drive flow mechanisms in microfluidics. The charged electrode creating non-neutral ionic layers between electrode–electrolyte surfaces is the main driving force of nonlinear flow generator platforms, actuated by electrokinetic forces, such as electro-osmosis and dielectrophoresis, which have been used for effective manipulation of the fluids inside the microfluidic chips.

Bengtsson K. et al. developed a compact electroosmotic pump, fabricated of a porous polycarbonate membrane and poly(3,4-ethylenedioxythiophene) polystyrene sulfonate (PEDOT: PSS) [122]. The designed pump can generate both high oscillating and steady flow rates up to ±400 µL/min. This pump can be easily connected to the most typical commercially available microfluidic chips.

Quite often, electrokinetic platforms represent an all-in-one chip, which combines a pumping technique, microfluidic channel network, and detecting system. The scientific group from Taiwan presented an on-chip CMOS-based low-power microfluidic pump, working on the principle of traveling-wave electroosmosis (TWEO) [123]. The flow-driven force of the developed microfluidic chip lay in the on-chip electrode array, consisting of 60 units. Each unit was built from four consecutive electrodes, which induce TWEO flow with the same phase shift in electrical potential. The authors have achieved low energy consumption of the developed device, which amounted to 1.74 mW, while achieving a 51 µm/s flow rate of diluted human serum.

Scott et al. presented an automated method for a challenge rarely discussed in the LOC literature—precise alignment of optics to a microchip electrophoresis channel combined with a centrifugal platform [124]. The proposed method does not require the presence of a fluorescence dye in the separation-sieving matrix but relies on the innate optical signatures. This means that this device does not require any additional hardware because the optical system is already employed for laser-induced fluorescence detection.

Chang-Ho Han and Jaesung Jang presented a microfluidic single-walled immunosensor equipped with electrohydrodynamic (EHD) focusing and DEP concentration for continuous and label-free detection of flowing *Staphylococcus aureus* [125]. The selectivity of the developed platform was tested against *Bacillus subtilis*, *Escherichia coli C3000*, and *Staphylococcus epidermidis* along with its sensitivity. Microchannels of the device with width/height: 200 µm/49 µm with two 1 mm diameter holes at the inlet and outlet reservoirs were manufactured, as was described in the work [126] and bonded onto the bio-functionalized sensor chip. The authors also used a syringe pump to infuse the bacterial solution into the microfluidic chip. The microfluidic immunosensor consists of three pairs of coplanar electrodes: focusing (10 µm gap), concentration (5 µm gap), and detection (20 µm gap), electrodes.

Zhu C. et al. presented an automated-by-CMOS ICs bio-sensing system with an AC–osmotic fluid-driven mechanism [127]. They combined an all-in-one fluid flow controller, bio-molecular sensor, cell manipulator, cytometer, and separator inside one chip. The system included a programable signal generator, which consists of a programable square wave generator, digital left shifter, high-voltage level shifter, and driver for dielectrophoresis electrodes. The output of the high-voltage driver was 5–9 V with fluid velocities reaching up to 160 µm/s.

#### Limitations

Electroosmotic-driven mechanisms are popular for their low power consumption, but they require more complex ways of forming flow-driven mechanisms. Such systems are often performed at one PCB: with all electronics and microfluidics channels placed on one surface, which makes device development very difficult.

### 3.4. Acoustic Streaming

Microfluidic devices are based on acoustic-driven methods that use sound waves to manipulate fluids inside microchannels. The key feature of this fluid-driven technology is its biocompatible and contactless nature, low-power consumption and cost-effectiveness, making it a great tool for biomedical and chemical research [128,129]. Generally, the driven force is the manipulation of the liquid achieved by the exchange of energy between acoustic waves and liquids.

In the following study, Ozcelik A. and Aslan Z. developed a simple pumping device for microfluidic applications, which is based on an acoustofluidics method [130]. The device consists of a glass capillary, 3D-printed fluid channels, top connector, and a piezoelectric transducer. Audio amplifier circuit generated sine waves with frequencies up to 70 kHz, which caused a flow rate up to 12 μL/min.

Chen X. et al. presented a novel approach for particle trapping, based on the acoustofluidic approach with smartphone-based detection mechanism [131]. The proposed system was tested for PSA detection in both the buffer and serum and achieved a low detection limit: 0.2 ng/mL, and a large dynamic response range of 0.3 to 10 ng/mL.

Zhao S. K. et al., developed a microfluidic platform for single-cell drug screening, based on the principle of tiny shear stress, placed on the cell induced by acoustic streaming [132]. This stress disorders the lipid structure of the cell membrane, which induces transient gaps between the lipid molecules, and after stress relief, the gaps disappear quickly, changing the cell’s permeability.

#### Limitations

These systems are similar to electro-osmosis systems and require complex structures placed on one PCB; moreover, they require acoustic converters, which enhance the power consumption of such systems.

Table 1 summarizes the most significant parameters of microfluidic systems with active flow control; it represents such parameters such as the liquid-driven method, flow rate, power characteristics, physical dimensions, presence of “off-chip” modules, and price.

## 4. Discussion

Table 2 summarizes all techniques of flow control, which were described in this review. We compared flow rates of the proposed devices, their advantages, and limitations, and mentioned typical application spheres.

Microfluidics is one of the fastest growing scientific fields. It can be applied practically in all spheres of life: pharmaceutical industry, diagnosis, chemical analysis, drug testing, healthcare, as control systems for environmental problems, and private testings. LOC and POC devices are one of the most significant applications of microfluidics, it has become more and more popular for its advantages: low cost, high testing speed, low reagent consumption, easiness of usage. Such devices can easily replace entire laboratories.

All these benefits cannot be fully achieved without different pumping techniques, applied for microfluidics. In this review, we have tried to consider the most promising techniques that exist at the moment. Here, we have described passive and active mechanisms for flow drive. We have discussed the working principle of these devices, tried to find the strongest and weakest parts of each technique.

Passive pumping mechanisms achieves low-cost easy-to-fabricate devices with simple principles of operation, which can be easily used for point-of-care applications. It is the simplest way used in autonomous microfluidic systems. Such systems become widespread among end users, which provide highly efficient analyses outside the laboratory. The main disadvantage of such systems is the limited range of tasks they can be applied in.

Active pumping mechanisms make up these disadvantages and present highly efficient, robust, fully automated, microfluidic flow regulatory systems. They are equipped with different pumps (electromagnetic, syringe, magnetic) and valves to control the fluid flow precisely. Such systems are often applied in the various spheres of life: foodborne testings, analysis, chemical reactions, biophysical assays, and cell cultivating. With the gradual modernization in the field of electronics, the size of such devices is rapidly decreasing, which in the near future, may lead to a complete replacement of passive flow control mechanisms in microfluidics.

Current developments in the field of microfluidics have led to the creation of compact all-in-one systems. We suppose that novel microfluidic systems regardless of the fluid flow control will be created to be as compact as possible. The authors also suppose that active flow control mechanisms will displace passive techniques and will combine all benefits of currently existing methods, which will lead to the creation of novel miniaturized high-precision all-in-one microfluidic systems without any external parts. We suppose that the current development of microelectronics will significantly reduce the dimensions of microfluidic devices and power consumption. This fact will lead to low-cost, portable, automated, highly accurate systems, which will provide a wide spectrum of experiments inside one chip.

## Figures and Tables

**Table 1:** Comparison of active pumping techniques for microfluidic applications.

Method	Power Supply	“Off-Chip” Elements	Type of MCU/PC	Method of Pressure Control	Feature	Price	Reference
Pressure-driven (Syringe pump)	10 V	Yes	Arduino-board, Raspberry Pi 2	PID-controller, Bang-bang method	Stability of the device to within ±1%	USD 110	[109]
Pressure-driven (Air/gas interception)	5 V, 7.7 W	No	Raspberry Pi 3 B+	Self-made program	All-in-one 3D-printed, based on gas interception system	EUR 340	[110]
Pressure-driven (Flow-focusing geometry)	6 V, 2 W	Yes	Atmega328P	-	Flow-focusing method for droplet generation	-	[111]
Pressure-driven (Dual-channel pressure pump)	3.7 V, 1330 mW	No	ESP-32 board	PID-controller	Wireless communication, sensitive PID controller, compactness	EUR 250	[112]
Pressure-driven (Pneumatic pressure controllers)	No information available	Yes	MCU	PID-controller	Cheap and highly accurate system	~USD 150	[113]
Centrifugal microfluidics (Portable bead-based platform)	No information available	Yes	Arduino-board	PID-controller	Combined centrifugal and heating modules serve as complete package for sample-to-answer analysis	~USD 250	[114]
Electroosmotic pump	13 mW	No	-	-	Low power consumption, low dimensions, high accurate control both of micro- and macroparticles	No information available	[122]
CMOS electroosmotic pump	1.74 mW	No	-	-	Very low power consumption, compact CMOS compatible process		[123]

**Table 2:** Comparison of different techniques for flow control in microfluidic devices.

Technique	Flow Rate	Advantages	Limitations	Typical Applications	Reference
**Passive Systems**					
Gravity-driven	µL/min	Stable pressure input and continuous fluid injection, rapid straightforward fluid driving	Too difficult to control pressure gradient between the inlet and outlet of the chip	Droplet generations systems, generating complex emulsions, bacteria enumeration, cell isolation	[71,74,75]
Capillary action	nL/min~µL/min	Stable flow rate, “auto-stop” mechanism, low consumption of the reagent, ultra-cheap fabrication	Complex structure, depends on the concentration of the liquid, channel surface needs to be modified by surfactant with multiple procedures, it is not easy to control the flow rate	Capillary pumps, autonomous capillary systems, POC-diagnostics, indicators for chemical reactions	[81,82,133,134,135]
Surface tension	µL/min	Low shear stress, high working period	Requires timely replenishment of the solution, complicated fabrication process	Long-term cultivating systems, droplet generation systems	[85,86,87,88]
Vacuum driven	nL/min	Compactness, combines driving of the fluid and reactions inside the chip	Requires specific vacuum storage, difficult to manufacture, disposable systems	Sample loading systems, POC diagnostics, air-bubble removal	[90,91,92,93,136]
Osmotic	µL/min	Continuous flow, which can last for more than a week	Unstable and inaccurate flow rate	Drug delivery systems, POC systems	[94,95,96]
Pressure-driven	µL/min~mL/min	User-friendly easy-to-use device, low-cost, accurate flow control	Human error—device, contains large components, not accurate flow rate, low repeatability	Cell separation, POC diagnostics, cell removal, droplet generation	[98,103,104,105,106]
**Active Systems**					
Pressure-driven	µL/min~mL/min	Easy-to-fabricate, easily accessible materials, simple working principle, high level of integration with computer	Dimensions of the system, unidirectional flow (syringe pumps), requires high accurate flow control systems	Cell separation, cell cultivation, diagnostics, droplet generation, autonomous systems	[109,110,111,112,113]
Centrifugal driven	µL/min~mL/min	Simultaneous multiple testings, simple working principle, biocompatible	Too complicated fabrication process	Cell enrichment, cell sorting, sample-to-answer assays, chemical lysis	[114,115,118,119,120]
Electrokinetic	µL/min	Effective highly accurate manipulation of the fluid, compactness, low power consumption	Too complicated fabrication and creation processes	Oscillating flow systems, POC systems	[122,123,127]
Acoustic	µL/min	Biocompatible, contactless nature of the device	Requires complex structure and expensive acoustic converters	Biomedical and chemical applications	[128,130,131,132]

## Data Availability

Not applicable.
